# Transcriptomic Analysis for the Identification of Metabolic Pathway Genes Related to Toluene Response in *Ardisia pusilla*

**DOI:** 10.3390/plants10051011

**Published:** 2021-05-19

**Authors:** Junping Xu, Chang Ho Ahn, Ju Young Shin, Pil Man Park, Hye Ryun An, Yae-Jin Kim, Su Young Lee

**Affiliations:** 1 Rural Development Administration, Floriculture Research Division, National Institute of Horticultural and Herbal Science, Wanju 55365, Korea; xujp0203@korea.kr (J.X.); ahnch3783@kiam.or.kr (C.H.A.); juyoung@korea.kr (J.Y.S.); pmpark@korea.kr (P.M.P.); hryun@korea.kr (H.R.A.); yj0503@korea.kr (Y.-J.K.); 2 Garden Plant Material Development Team, Korea Institute of Arboretum Management, Sejong 30106, Korea

**Keywords:** transcriptomic analysis, RNA-seq, toluene, *Ardisia*, DEG

## Abstract

Toluene is an industrial raw material and solvent that can be found abundantly in our daily life products. The amount of toluene vapor is one of the most important measurements for evaluating air quality. The evaluation of toluene scavenging ability of different plants has been reported, but the mechanism of plant response to toluene is only partially understood. In this study, we performed RNA sequencing (RNA-seq) analysis to detect differential gene expression in toluene-treated and untreated leaves of *Ardisia* *pusilla*. A total of 88,444 unigenes were identified by RNA-seq analysis, of which 49,623 were successfully annotated and 4101 were differentially expressed. Gene ontology analysis revealed several subcategories of genes related to toluene response, including cell part, cellular process, organelle, and metabolic processes. We mapped the main metabolic pathways of genes related to toluene response and found that the differentially expressed genes were mainly involved in glycolysis/gluconeogenesis, starch and sucrose metabolism, glycerophospholipid metabolism, carotenoid biosynthesis, phenylpropanoid biosynthesis, and flavonoid biosynthesis. In addition, 53 transcription factors belonging to 13 transcription factor families were identified. We verified 10 differentially expressed genes related to metabolic pathways using quantitative real-time PCR and found that the results of RNA-seq were positively correlated with them, indicating that the transcriptome data were reliable. This study provides insights into the metabolic pathways involved in toluene response in plants.

## 1. Introduction

As a toxic volatile organic solvent, toluene is widely used in the production of daily life products, such as gasoline mixtures, benzene, cosmetics, ink, adhesives, paints, coatings, and glues [1]. It is difficult to degrade toluene by ultraviolet rays or atmospheric oxidation [2]. With the increase in population and development of cities, people stay indoors longer, and interior air quality has become more important. Toluene is easily absorbed by humans through the respiratory tract because of its widespread use at the individual and industrial levels [3]. In addition, the concentration of toluene in indoor air is usually higher than that in outdoor air, which makes it inevitable for humans to be exposed to toluene [4]. Toluene is hazardous to human health. Both short-term exposure to high concentrations or long-term exposure to low concentrations of toluene may cause serious health-related issues, such as dizziness, nausea, vomiting, loss of appetite, dermatitis, lung injury, and damage to the central nervous and immune systems, and it may even cause death in severe cases [5,6,7].

Ornamental plants are receiving greater attention for indoor purification of air because they are economically viable and environmentally friendly. To date, the ability of many plants to specifically remove indoor harmful gases has been studied [8,9]. In these studies, the purification capacity of different plants for toluene has also been investigated [8,10,11,12]. Among the 12 plant species tested, *Sansevieria trifasciata* has been found to have the highest toluene removal capacity [12]. However, little is known about the toluene metabolism pathway in plants. Therefore, there is an urgent need to understand the metabolic response mechanism of plants to toluene and develop new germplasms with the ability to enhance toluene metabolism. These new germplasms can be used as purification plants to improve air quality economically and effectively.

*Ardisia* is a flowering plant in the subfamily Myrsinoideae. At present, more than 700 species of this plant have been reported, located mainly in the tropics [13]. *Ardisia pusilla* (*A. pusilla*) is a shrub with light green leaves, white flowers, and red berries. It is a popular indoor ornamental plant in Korea [14]. According to Song, the removal efficiency of toluene by *A. pusilla* is significantly higher than that of other plants [15]. Ahn et al. overexpressed the *AtNDPK2* gene in *A. pusilla* to further improve its ability to absorb toluene [14]. Previous studies on the *AtNDPK2* gene have mainly reported that it can improve abiotic stress tolerance in plants [11,16,17,18].

As a high-throughput next-generation sequencing (NGS) technology, RNA sequencing (RNA-seq) has developed rapidly and has become an indispensable tool for analyzing differential gene expression and gene regulatory networks in transcriptome research. Particularly for atypical plants, even if there is no reference genome, the genome can be constructed by de novo transcriptome assembly [19]. Chen et al. revealed the metabolic defense mechanism of rice against predominant polybrominated diphenyl ether (PBDE) pollution by performing RNA-seq on rice grown to maturity in soil containing PBDE [20]. To date, RNA-seq has not been used to study the metabolic defense mechanisms of plants against toxic volatile organic compounds.

In this study, we performed RNA-seq analysis using Illumina NovaSeq 6000 (Illumina, San Diego, CA, USA) to conduct an in-depth sequencing of *A. pusilla* after toluene treatment, followed by comparison of toluene-treated and untreated plants. To understand the reactive response mechanism of plants to toluene exposure and the metabolic pathway of toluene in plants, we analyzed the differential gene expression of the transcriptome and verified it by quantitative real-time PCR (qRT-PCR). We also examined the gene expression related to toluene metabolism in *AtNDPK2*-transgenic *A. pusilla* plants with enhanced toluene absorption capacity.

## 2. Results

### 2.1. Illumina Sequencing and De Novo Assembly

To gain additional insight into the transcriptomic response of *A. pusilla* to toluene treatment, the leaves of the toluene-treated (1-1, 1-2) and untreated groups (2-1, 2-2) were chosen to construct cDNA libraries. Four cDNA libraries were generated containing 135.59, 162.62, 142.96, and 136.26 million clean reads, respectively (Table 1). GC contents in the four libraries were 50.3%, 49.99%, 50.54%, and 49.37%, whereas Q30 percentage (% of bases with quality over phred score 30) was 96.03%, 95.69%, 95.78%, and 95.87%, respectively. Trimmed reads for each sample were aligned to the assembled reference using the Bowtie program [21]. For DEG analysis, the abundance of unigenes across the samples was estimated into read count as an expression measure using the RSEM algorithm [22]. Table 1 shows the overall read mapping ratio for each sample; 1-1, 1-2, 2-1, and 2-2 had 74.33%, 72.08%, 76.99%, and 71.92% of the mapped sequencing reads, respectively, obtained after aligning to the assembled reference genome.

Trimmed reads for every sample were merged into one file to construct a combined reference. Since there were no genomic sequences of *A. pusilla*, the merged data were assembled into transcript contigs using Trinity software. The statistics of the assembled transcript contigs are listed in Table S2. The sequence reads were assembled into 99,322 contigs covering 93,764,604 bases, with N50 values of 1137 bp and an average contig length of 694.06 bp (Table S2). The CD-HIT-EST program (http://weizhongli-lab.org/cd-hit, accessed on 22 February 2020) was used to filter and cluster 88,444 unigenes from the longest contigs.

### 2.2. Annotation Results

**Figure 1:**
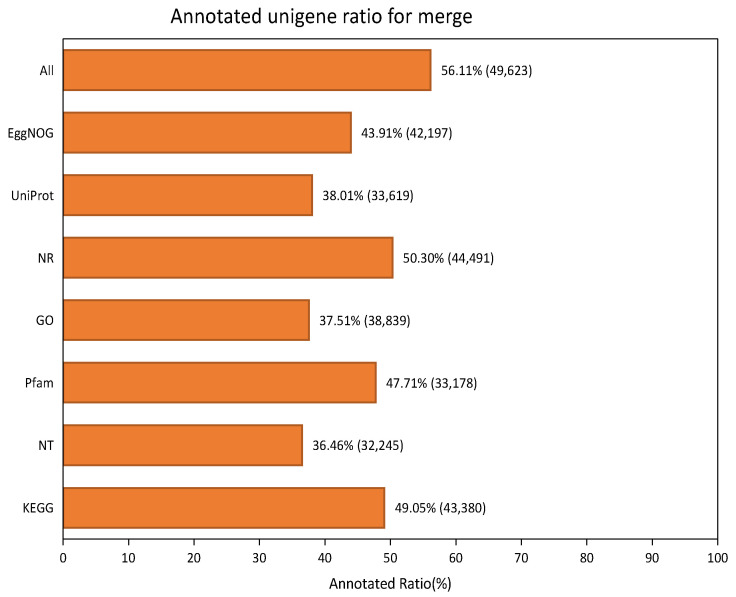
Annotated result of *A. pusilla* leaf transcriptome using various databases.

For functional annotation, 49,623 (56.11%) of the 88,444 unigenes in *A. pusilla* leaf transcriptome were successfully annotated by searching EggNOG, UniProt, NR, GO, Pfam, NT, and KEGG functional databases using BLASTX or BLASTN with an E-value cut-off of 1.0E-5. Among them, 44,491 (50.3%), 43,380 (49.05%), 33,178 (47.71%), 42,197 (43.91%), 33,619 (38.01%), 38,839 (37.51%), and 32,245 (36.46%) unigenes were matched with the NR, KEGG, Pfam, EggNOG, UniProt, GO, and NT databases, respectively (Figure 1).

### 2.3. Annotation on GO Database

**Figure 2:**
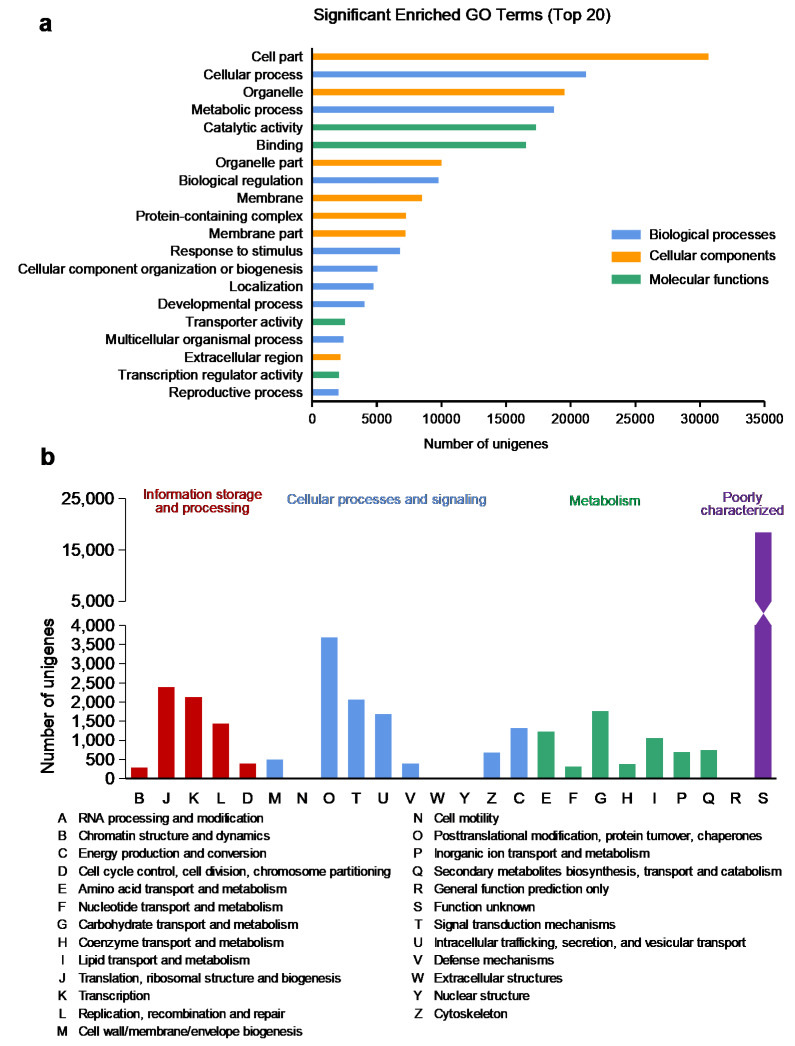
Functional annotation of unigenes identified in *A. pusilla* leaf transcriptome. (**a**) GO functional classification analysis; (**b**) EggNOG classification analysis.

The GO database was used to classify the annotated unigenes for functional annotation of the *A. pusilla* leaf transcriptome. The GO terms belonging to BP, CC, and MF were identified. There were 52 subcategories related to BP, including representative subcategories as the “cellular process” (21,167 unigenes) and “metabolic process” (18,724 unigenes). There were 26 subcategories related to CC, mainly including the “cellular part” (30,669 unigenes) and “organelle” (19,538 unigenes). There were 16 subcategories related to MF, which were mainly involved in “catalytic activity” (17,323 unigenes) and “binding” (16,558 unigenes) (Figure 2a and: Table S3). The results revealed that most of the identified genes were involved in the “cellular part” and “metabolic process” in the response of *A. pusilla* to toluene.

### 2.4. Annotation on EggNOG Database

To identify the proteins distributed in eukaryotic orthologous groups, clusters of orthologous groups and non-supervised orthologous groups, the annotated unigenes were mapped to the annotation of the corresponding orthologous groups in the EggNOG database. For EggNOG annotation, 42,197 unigenes were divided into 25 EggNOG categories. Some of the unigenes were assigned to more than one category. The largest proportion of unigenes belonged to “undefined function” (44.66%), followed by “posttranslational modification, protein turnover, chaperones” (8.7%), “translation, ribosomal structure and biogenesis” (5.65%), “transcription” (5.04%), and “signal transduction mechanisms” (4.88%) (Figure 2b). In particular, “secondary metabolites biosynthesis, transport and catabolism” category accounted for 1.77% of total annotated unigenes by EggNOG (Figure 2b), and this category was further analyzed for its role in the metabolic pathway underlying toluene response in *A. pusilla* plants.

### 2.5. DEG Analysis Results

Based on the results of the annotation analysis, DEGs were analyzed, and significant results were selected according to the following conditions: |fc| ≥ 2 and *p*-value < 0.05. Comparing the transcription profiles of toluene-treated and untreated groups, 4101 DEGs were obtained, of which 2100 were upregulated and 2001 were downregulated (Table 2).

GO analysis classified 2854 DEGs into three functional categories: BP, CC, and MF (Table 2, Figure S1). The number of upregulated DEGs (1456) was similar to that of downregulated DEGs (1398) (Table 2). GO analysis demonstrated that a large number of GO subcategories, such as cellular processes (1504), metabolic processes (1340), biological regulation (809), and response to stimulus (626), were the most enriched categories in BP (Figure S1). The highest number of DEGs involved in CC belonged to cell part (2253), organelle (1454), membrane (675), and organelle part (584) (Figure S1). In addition, in terms of MF, catalytic activity (1245), binding (1130), transcription regulator activity (229), and structural molecular activity (194) were the most enriched subcategories (Figure S1).

**Figure 3:**
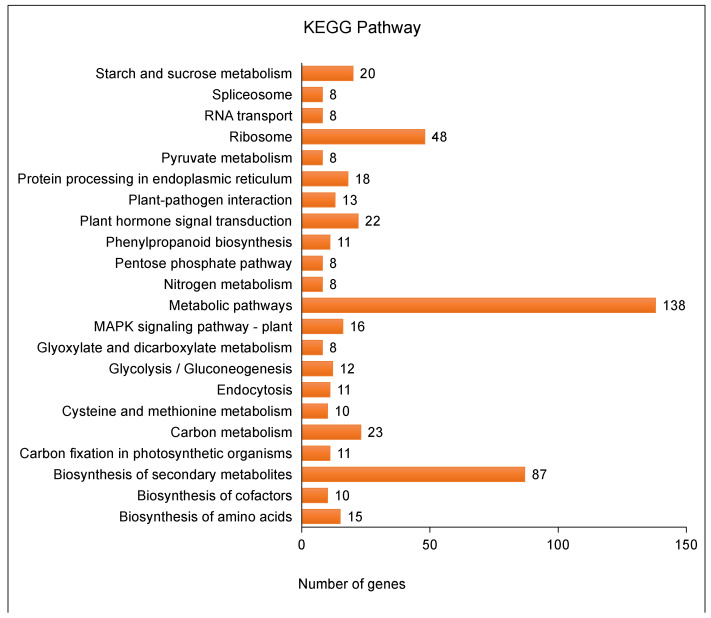
KEGG pathway analysis of DEGs in toluene treated and untreated *A. pusilla*.

To identify different pathways of activation in *A. pusilla* plants after toluene treatment, we analyzed the KEGG pathway of all DEGs, in which 1610 genes were upregulated and 1491 genes were downregulated (Table 2). In this study, DEGs were mapped to 95 pathways (data not shown), and the top 20 major pathways are listed in Figure 3. In addition, the metabolic pathway, biosynthesis of secondary metabolites, ribosome, and carbon metabolic pathways had the highest number of DEGs between toluene-treated and untreated *A. pusilla* plants (Figure 3). Interestingly, all 48 genes of the ribosome pathway were upregulated (Figure S2). A total of 11 genes involved in endocytosis were downregulated (Figure S3). In particular, we focused on the AT4G09320 gene (*NDPK1*) of the MAPK signaling pathway. The expression of *NDPK1* in the toluene-treated group was 23.2-fold higher than that in the untreated group (Additional file 1: Table S4). A previous study also reported that *NDPK2* transgenic *A. pusilla* plants show significantly increased toluene uptake compared to non-transgenic plants [14].

### 2.6. Transcriptional Factors (TFs)

**Figure 4:**
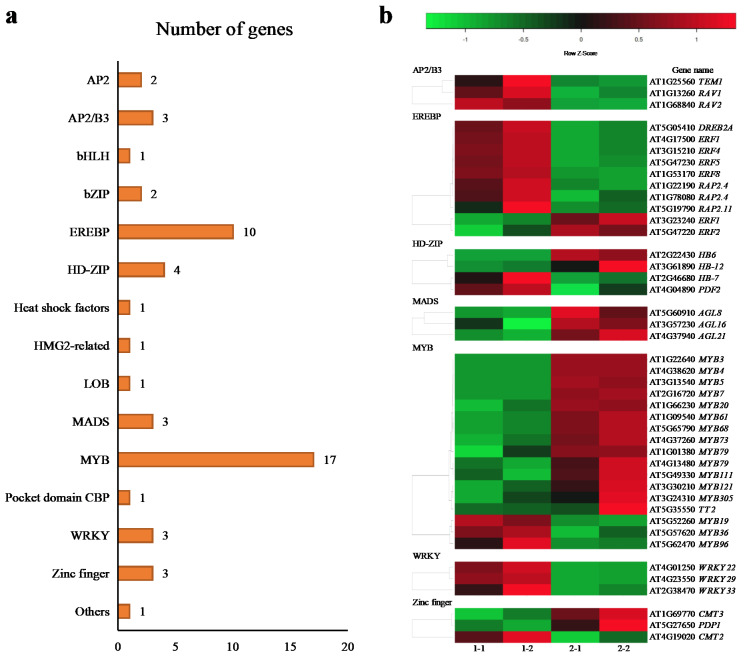
Transcriptional factors of DEGs identified in toluene untreated and treated *A. pusilla*. (**a**) The present study identified 53 TFs that were classified into 14 transcription factor families; (**b**) heat maps of various types TFs in toluene untreated (1-1, 1-2) and treated (2-1, 2-2) *A. pusilla*. Heatmap color represents the expression level of each gene (rows) in different treatments. (fold-change > 2, *p*-value < 0.05). Red bars: upregulation; green bars: downregulation.

Among the DEGs, 53 genes encoding TFs belonging to 14 TF families were identified (Figure 4a), which included AP2/B3 (three downregulated), EREBP (two upregulated and eight downregulated), HD-Zip (two upregulated and two downregulated), MADS box (three upregulated), MYB (14 upregulated and 3 downregulated), WRKY (three downregulated), and zinc finger (two upregulated and one downregulated) (Figure 4b).

### 2.7. Toluene Metabolic Pathway

**Figure 5:**
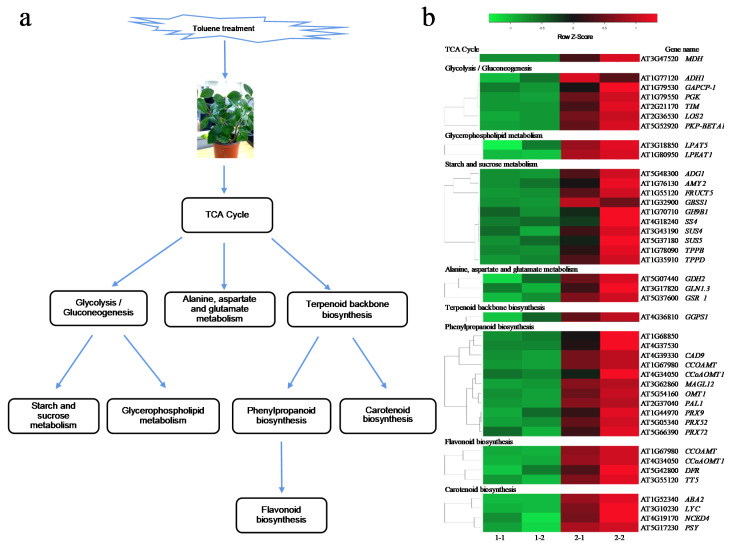
The models of toluene metabolic mechanism in *A. pusilla*. (**a**) Toluene response related to metabolic defense pathways in *A. pusilla*; (**b**) heat maps of metabolic pathways DEGs in toluene untreated (1-1, 1-2) and treated (2-1, 2-2) *A. pusilla*. Heatmap color represents the expression level of each gene (rows) in different treatments. (fold-change ≥ 2, *p*-value < 0.05). Red bars: upregulation; green bars: downregulation.

Based on the KEGG analysis of DEGs, we constructed a metabolic pathway for toluene in *A. pusilla* plants (Figure 5a). Our results revealed that the expression of the malate dehydrogenase gene (*MDH*) of the tricarboxylic acid cycle (TCA cycle) was upregulated, indicating that the production of oxaloacetate and phosphoenolpyruvate had increased. Phosphoenolpyruvate is catalyzed by enolase (encoded by *LOS2*) to form 2-phosphoglycerate, which enters glycerophospholipid metabolism and starch and sucrose metabolism through glycolysis and glycolysis, respectively. In addition, phosphoenolpyruvate is catalyzed by pyruvate kinase (encoded by *GGPS1*) to form pyruvate, which enters carotenoid, phenylpropanoid, and flavonoid biosynthesis through terpenoid backbone biosynthesis (Figure 5a,b). Moreover, the expression of three genes, *GDH2*, *GLN1.3*, and *GSR_1*, involved in alanine, aspartate, and glutamate metabolism, respectively, was upregulated (Figure 5b).

### 2.8. Validation of RNA-Seq Results by qRT-PCR

**Figure 6:**
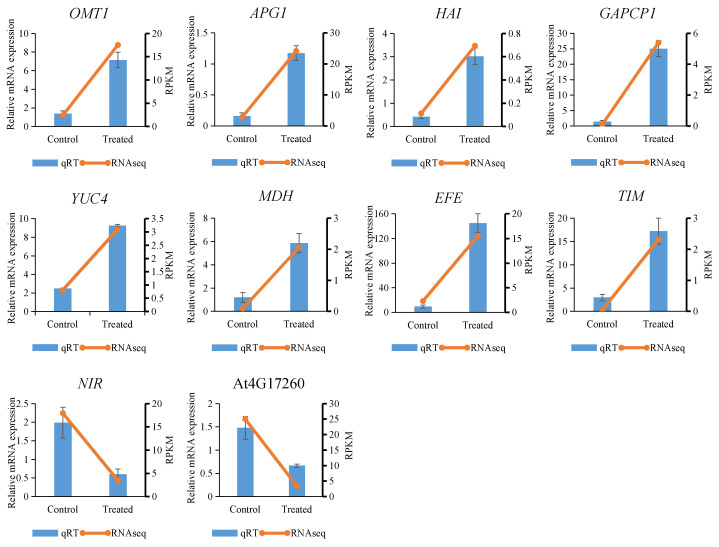
The gene expression analysis of metabolic pathway genes in toluene treated and untreated *A. pusilla* plants. Values are means of three biological repeats. Error bars indicate ± SE. OMT1: O-methyltransferase 1; APG1: S-adenosyl-l-methylionine-dependent methyltransferases superfamily protein; HAI: H [+]—ATPase 1; GAPCP1: glyceraldehyde-3-phosphate dehydrogenation of plastid 1; YUC4: flavin binding monooxygenase family protein; MDH: malate dehydrogenase; EFE: ethylene forming enzyme; TIM: triosephosphate isomerase; NIR: nitrite reductase 1; AT4G17260: lactate/malate dehydrogenase family protein.

To verify the reliability of the RNAseq results, ten genes related to the metabolic pathway were randomly selected for qRT-PCR analysis, including eight upregulated genes and two downregulated genes (Table S1). The relative expression changes in qRT-PCR results were largely consistent with the RNA-seq data. When we compared the toluene-treated group to the untreated group, the expression of *OMT1*, *APG1*, *HAI*, *GAPCP1*, *YUC4*, *MDH*, *EFE*, and *TIM* genes was upregulated by 5.2-, 7.4-, 7.2-, 16.9-, 3.8-, 4.9-, 15.6-, and 5.8-fold, respectively. These results are approximately consistent with the RNA-seq analysis results showing 8.1-, 8.9-, 6.1-, 57.0-, 4.4-, 25.4-, 7.7-, and 59.4-fold upregulation (Figure 6). In RNA-seq analysis, two downregulated genes, NIR −4.7-fold) and AT4G17260 (−6.6-fold) were detected with similar trends to those revealed by qRT-PCR analysis with −2.9-fold and −2.2-fold downregulation, respectively (Figure 6).

### 2.9. Gene Expression Analysis Related to Toluene Purification in AtNDPK2-Transgenic A. pusilla Plants

**Figure 7:**
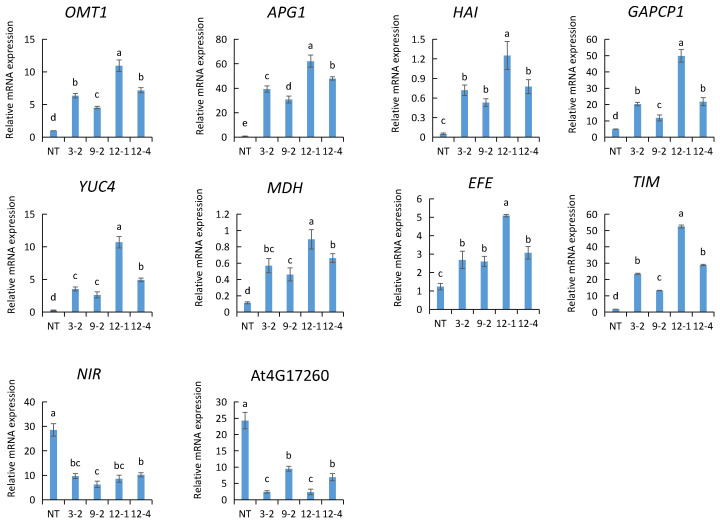
The expression analysis of metabolic genes in toluene treated and untreated AtNDPK2- transgenic *A. pusilla*. NT: non-transgenic *A. pusilla* plants; 3-2, 9-2, 12-1, 12-4: AtNDPK2-transgenic *A. pusilla* lines. Values are means of three biological repeats. Error bars indicate + SE. Different small letters indicated a significant difference (*p* < 0.05, Duncan’s multiple range test).

Our previous study showed that the detoxification ability of *AtNDPK2*-transgenic *A. pusilla* plants is enhanced by the exogenous application of toluene [14]. In the current study, the results of DEG analysis showed that *AtNDPK1* expression was upregulated in toluene-treated *A. pusilla*. *AtNDPK1*- and *AtNDPK2*-coded proteins displayed 57% sequence identity. We speculated that the increase in NDPK expression may contribute to the absorption and metabolism of toluene in plants. The expression of genes related to the toluene metabolic pathway was detected in the *NDPK2* transgenic plants by qRT-PCR. The expression of *OMT1*, *APG1*, *HAI*, *GAPCP1*, *YUC4*, *MDH*, *EFE*, and *TIM* genes in four *AtNDPK2*-transgenic plants was considerably higher than that in non-transgenic plants. In addition, the expression of *NIR* and *AT4G17260* was higher in the non-transgenic plants than in transgenic plants. These results indicate that the introduction of *AtNDPK2* could be helpful in increasing or decreasing the expression of these genes (Figure 7). RNA-seq analysis and qRT-PCR validation results showed that these genes were associated with toluene metabolism.

## 3. Discussion

In recent years, RNA-seq has become a powerful technology for understanding various molecular mechanisms and solving several biological problems. Even if there is no reference genome available, the short reads generated by Illumina sequencing can be assembled into contigs, and the DEGs can be identified by de novo transcriptome assembly [10].

In this study, we treated *A. pusilla* plants with toluene, followed by RNA-seq analysis of the treated and untreated leaves with Illumina, to obtain a large amount of cDNA sequence data, which provides a basis for the identification of DEGs and a more detailed study of the toluene metabolic pathway in plants. Based on Illumina RNA-seq data, each sample produced more than 14 GB of clean read segments, which were assembled into 88,444 unigenes with an average length of 637 bp and a median size of 1049 bp. Through BLASTX or BLASTN protein database annotation, including that of EggNOG, UniProt, NR, GO, Pfam, NT, and KEGG functional databases, 49,623 unigenes (56.11%) were successfully compared to known proteins, and 43.89% of the unigenes showed no similarity, indicating that more genetic data are required for annotation. GO is an international standard classification system for gene function [23]. It provides dynamic, controlled terminology, and strictly defined concepts to comprehensively describe the characteristics of genes and their products in any organism. Moreover, GO classification can provide an opportunity to understand the distribution of gene function at the macro level and predict the physiological function of each unigene [24]. In the present study, according to GO analysis, the most involved gene subcategory was “cell part,” followed by cellular process, organelle, and metabolic process. DEGs were also distributed among the four gene sub-categories. There are nearly 90 reference metabolic pathways in the KEGG database, which is very helpful for researchers to study metabolic and regulatory processes [25]. Through the analysis of the KEGG enrichment pathway, 182 DEGs were identified, which helped us elucidate the toluene response metabolic pathway.

Our study aimed to explore the metabolic pathways of toluene response in *A. pusilla* plants by RNA-seq and qRT-PCR analyses. Experiments on labeled (14C) aromatic hydrocarbons have shown that after plants absorb toluene, the aromatic ring of toluene is cleaved during its metabolic transformation [26,27]. Ugrekhelidze et al. speculated that the oxidative cleavage of toluene could occur through two ways: oxidation of a methyl group to a carboxyl group, followed by ring hydroxylation to produce α-carboxymucic acid, and ring hydroxylation of toluene without preliminary oxidation of the methyl group to produce α-methylmucic acid [28]. Our results showed that the expression of *MDH* associated with the TCA cycle was significantly upregulated. This is consistent with the high radioactivity levels of malic and oxalic compounds reported previously by Ugrekhelidze et al. [28]. We also found that a large number of genes were upregulated in glycolysis/gluconeogenesis, starch and sucrose metabolism, glycerophospholipid metabolism, carotenoid biosynthesis, phenylpropanoid biosynthesis pathway, and flavonoid biosynthesis. Based on the above results, we constructed a comprehensive metabolic pathway map, clearly explaining the metabolic pathway of toluene (Figure 5a,b). Finally, we verified 10 genes in the metabolic pathway by qRT-PCR, which proved the reliability of the RNA-seq results.

Transcriptional regulation of gene expression depends on the recognition of promoter elements by TFs. In the past few years, several TFs have been identified in plants [29]. TFs involved in toluene metabolism in plants have not yet been reported. It is necessary to analyze the expression levels of TFs to understand their roles in toluene metabolism. In the present study, 53 TF-coding DEGs, associated with the most representative TF families (MYB, EREBP, AP2/B3, and WRKY), were identified. TFs play important roles in plant development and responses to the environment [30]. In the past decade, extensive studies have been carried out on R2R3-MYB-coding genes, and it has been found that the MYB family is large and has diverse functions, participating in various biological functions, including responses to biotic and abiotic stresses, development, differentiation, metabolism, and defense. Some examples include phenylpropanoid biosynthesis [31], carotenoid biosynthesis [32,33], and flavonoid biosynthesis [34,35]. *MYB20* TF regulates phenylalanine biosynthesis [36], and *MYB96* can activate *Arabidopsis* cuticle wax biosynthesis [37]. The expression of *MYB121* in *Arabidopsis* is upregulated after exposure to formaldehyde [38]. In the present study, after *A. pusilla* plants were treated with toluene, RNA-seq analysis showed that the 14 genes encoding TFs of the MYB family were upregulated and three genes were downregulated (Figure 4). These results indicate that some TF-coding genes of the MYB family play important roles in toluene metabolism.

When treated with toluene, 8 genes out of 10 DEGs of the EREBP family were upregulated (Figure 4b), which is a member of the plant-specific TF family. Previous studies have shown that *AtERF4*, *AtERF5*, *AtERF8*, and *AtDREB2A* are negative regulators that can inhibit gene transcription, and *AtERF5* may play an important role in plant innate immunity by coordinating chitin and other defense pathways [39,40,41,42,43]. The present study showed that the expression levels of *AtERF4* (AT3G15210), *AtERF5* (AT5G47230), *AtERF8* (AT1G53170), and *AtDREB2A* (AT5G05410) were downregulated during toluene metabolism. The expression levels of *AtERF-1* (AT4G17500), *AtERF2* (AT5G47220), *AtERF3*, and *AtRAP2.4* (AT1G78080, AT1G22190) were also downregulated. However, *RAP2.11* (AT5G19790) and *ERF1* (AT3G23240) expression was upregulated to activate the ethylene response.

The expression of AP2/B3 TF family-coding genes (*TEM1*, *RAV1*, and *RAV2*) and WRKY TF family-coding genes (*WRKY22*, *WRKY29*, and *WRKY33*) were also downregulated (Figure 4b). *TEM1* controls flower senescence and abscission [44]. *Arabidopsis TEM1* and *TEM2* double mutants are more tolerant than plants overexpressing *TEM* under conditions of increasing soil salinity [45]. Similarly, *RAV1* and *RAV2* are multifunctional negative regulators of plant growth, abiotic stress, drought, and salt stress [46]. This is consistent with our results; the expression of these genes was downregulated during toluene response, which may be negative regulators. WRKY TFs play an important regulatory role in various stress adaptations. *WRKY22* and its homolog, *WRKY29*, are part of class IIE WRKYs, which are markers of pathogen-triggered immunity [47]. *WRKY33* is related to the ROS detoxification mechanism, and overexpression of *WRKY33* can improve *Arabidopsis* tolerance to salt stress [48]. In our results, we determined that *WRKY22*, *WRKY29*, and *WRKY33* were highly sensitive to toluene treatment and that their expression was downregulated.

Nucleoside diphosphate kinase (*NDPK*) is a highly conserved multifunctional protein that is mainly involved in maintaining the balance between NDP and NTP, and it can regulate various eukaryotic cell activities, including cell proliferation, development, and differentiation [49]. At present, five *NDPK*-coding genes have been identified in plant species, namely, *NDPK1* to *NDPK5*. The roles of *NDPK1*, *NDPK2*, and *NDPK3* in signal transduction and oxidative stress have been demonstrated [50,51]. RNA-seq analysis in the current study showed that the expression of *NDPK1* increased after toluene treatment. We speculate that *NDPK1* plays an important role in toluene detoxification and metabolism. Previous studies have shown that the overexpression of *NDPK2* enhances tolerance to various abiotic stresses [17,18], especially in favor of the detoxification ability of *A. pusilla* to toluene [14]. To verify whether the overexpression of *AtNDPK2* affects the expression of other genes related to toluene reaction or metabolism, we used qRT-PCR to detect the expression of related DEGs after toluene treatment. The results showed that the overexpression of *NDPK2* promoted or decreased the expression of genes related to toluene reaction (Figure 7).

## 4. Materials and Methods

### 4.1. Plant Materials and Toluene Treatment Conditions

*A. pusilla* plants were collected from the Floriculture Research Division, National Institute of Horticultural and Herbal Science, Rural Development Administration, Wanju 55365, South Korea. *A. pusilla* plants (20 cm height) grown in a greenhouse were placed in a sealed chamber at room temperature (25 ± 5 °C) under a pressure of 760 mm Hg with 12/12 h light/dark cycle. After that, toluene treatment was applied at a concentration of 1.5 ppm inside the chamber for 5 h. *A. pusilla* plants without toluene treatment were used as controls. The leaves of three plants in each of the toluene treatment group and the control group were collected, and immediately frozen in liquid nitrogen. This process was repeated twice, and all leaves were stored at −80 °C until further analysis.

### 4.2. RNA Extraction

*A. pusilla* leaf samples were ground into powder in a mortar with liquid nitrogen, and total RNA was extracted from the samples using TRIzol reagent (MRC, Cincinnati, OH, USA) according to the manufacturer’s protocol. The quality and quantity of RNA were assessed using a spectra max quick drop microvolume spectrophotometer (Molecular Devices, San Jose, CA, USA) and analyzed on 1% agarose gel. The transcriptome sequencing libraries were prepared by mixing equal quantities of RNA from the samples [14].

### 4.3. Deep Sequencing

Two replicates of each toluene-treated and untreated RNA samples were sent to Macrogen Inc. (Seoul, Korea) for deep sequencing and generation of datasets. TruSeq Stranded mRNA LT Sample Prep Kit (Illumina, San Diego, CA, USA) was used to prepare a cDNA library according to the TruSeq Stranded mRNA Sample Preparation Guide, Part # 15031047 Rev. E. Briefly, DNase was used to remove DNA contamination, then the poly-A mRNAs were purified, and the mRNA was disintegrated into short fragments. Cleaved RNA fragments were synthesized into cDNA using random hexamer primers. After purification, the cDNA fragments were ligated to universal adapters containing sequencing priming sites. Subsequently, fragments were amplified using PCR and analyzed by agarose gel electrophoresis, and fragments with insert sizes between 200 and 400 bp were subjected to deep sequencing. For paired-end sequencing, both ends of the cDNA were sequenced using an Illumina NovaSeq 6000 (Illumina, San Diego, CA, USA).

### 4.4. Sequence Data Analysis and De Novo Assembly

To analyze the quality control of the sequenced raw reads, overall read quality, total bases, total reads, GC (%), and basic statistics were calculated. In order to reduce biases in analysis, artifacts such as low-quality reads, adaptor sequences, contaminant DNA, or PCR duplicates were removed using the Trimmomatic program [52]. The sliding window method was used, and bases of reads that did not qualify inside the window size 4, and mean quality 15 were trimmed [52]. Afterwards, reads with lengths shorter than 36 bp were dropped to produce trimmed data. The trimmed reads for all samples were merged into one file to perform transcriptome assembly. Subsequently, merged data were assembled using Trinity software (https://github.com/trinityrnaseq/trinityrnaseq/wiki) [53], which is generally utilized for de novo reconstruction of transcriptomes and combining read sequences of a certain length of overlap to form longer fragments without N gaps, called contigs. For assembled genes, the longest contig of the assembled contigs was filtered and clustered into non-redundant transcripts using the CD-HIT-EST program [54,55]. We defined these transcripts as unigenes. The unigenes were used for subsequent annotation and further differentially expressed gene (DEG) analysis.

### 4.5. Sequence Annotation and Classification

For annotation, unigenes were searched against various public databases such as NCBI Nucleotide (NT, https://www.ncbi.nlm.nih.gov/nucleotide/), National Center of Biotechnology Information (NCBI) non-redundant protein (NR, https://www.ncbi.nlm.nih.gov/protein/), Kyoto Encyclopedia of Genes and Genomes (KEGG) (http://www.genome.jp/kegg/ko.html), Pfam (https://pfam.xfam.org/), Gene Ontology (GO, http://www.geneontology.org/), UniProt (http://www.uniprot.org/), and EggNOG (http://eggnogdb.embl.de/) using BLASTN of NCBI BLAST and BLASTX of DIAMOND software with an E-value default cutoff at 1.0E-5 [56]. The abundance of unigenes across samples was estimated using the RSEM algorithm [22,57]. The expression level was calculated using the read count. This value was used for further DEG analyses. ORF prediction for unigenes was performed using the TransDecoder program to identify candidate coding regions within transcript sequences [58].

### 4.6. Identification of DEGs

The read count values of contigs obtained through RSEM were used as the original raw data. Low-quality transcripts were filtered during data preprocessing. Subsequently, trimmed mean of M-values (TMM) normalization was performed, and statistical analysis was performed using fold change, exact test using edgeR per comparison pair [58]. Significant results were selected on the conditions of |fc| ≥ 2 and *p*-value < 0.05. For significant lists, hierarchical clustering analysis was performed to group similar samples and contigs. These results are graphically depicted using a heatmap and dendrogram. Classification of GO terms was subsequently performed using an in-house script [59]. The GO terms belonging to biological process (BP), cellular component (CC), and molecular function (MF) are listed. For KEGG pathway analysis of the DEGs, bidirectional best hit (BBH) was used to search against the KEGG database to obtain the KO (reference pathway) number of the KEGG annotations [60].

### 4.7. Validation of RNA-Seq Analysis by qRT-PCR

To validate the transcriptome-based DEGs results, 10 DEGs related to metabolic pathways were selected and examined by qRT-PCR using actin as a housekeeping gene. Primers were designed using Primer 3 software (www.embnet.sk/cgi-bin/primer3_www.cgi). The primer names and sequences used for primer design are listed in Table S1. In detail, the first-strand cDNA was used as the template for each reaction and 2 μL of cDNA (1:9 diluted) was placed in a 20 μL reaction mixture containing 10 μL of iQ™ SYBR-Green Supermix (2×) and 0.2 μL of each primer (10 pmol). The qRT-PCR was run on a CFX96 qPCR-PCR machine (Bio-Rad, Hercules, CA, USA), and the thermal cycling conditions were adjusted as follows: 95 °C for 3 min, followed by 40 cycles of 95 °C for 10 s and 63 °C for 30 s. Actin was used as the reference gene to normalize the gene expression level, and relative gene expression was calculated using the Ct (2^−ΔΔCt^) method. The above experiment was repeated three times.

### 4.8. Gene Expression Analysis Related to Toluene Purification in AtNDPK2-Transgenic A. pusilla Lines

To further analyze the metabolic pathways of plants in the process of toluene absorption and decomposition, four transgenic *A. pusilla* plants with *AtNDPK2* obtained previously in our laboratory were used along with non-transgenic *A. pusilla* as a control [14]. Briefly, the plants were transferred into pots (20 cm in diameter) containing autoclaved soil and placed inside sealable plastic bags. The bags were sealed and placed in the culture room. After one week, the bags were partially opened, and two weeks later, they were fully opened. In the third week, the plants were cultured under ambient greenhouse conditions. Two months later, the surviving plants were placed in a sealed chamber at room temperature (25 ± 5 °C) under a pressure of 760 mm Hg with 12/12 h light/dark cycle. After toluene treatment was carried out at a concentration of 1.5 ppm inside the chamber for 5 h, the leaves of three plants in each line were mixed and immediately frozen in liquid nitrogen for further analysis. RNA extraction, cDNA synthesis, and qRT-PCR were performed as described above. The differential expression of metabolic genes related to toluene absorption and decomposition in transgenic *A. pusilla* lines was analyzed. The experiment was repeated three times.

## 5. Conclusions

In conclusion, we performed RNA-seq analysis and screened 4101 DEGs from *A. pusilla* plants after toluene treatment. The results of cluster analysis and functional annotation of DEGs enabled us to further investigate the metabolic pathway of toluene in *A. pusilla* plants. Toluene first enters the TCA cycle through ring cleavage and oxidation and then approaches other metabolic pathways, such as glycolysis/gluconeogenesis; starch and sucrose metabolism; glycerophospholipid metabolism, alanine, aspartate, and glutamate metabolism; terpenoid backbone biosynthesis; phenylpropanoid biosynthesis; flavonoid biosynthesis; and carotenoid biosynthesis. Our study provides insights into the metabolic mechanism underlying toluene response in plants. The identification of DEGs provides us with new genetic resource information, which would be helpful in developing plants for toluene air pollution remediation. In addition, qRT-PCR data supported that the overexpression of *AtNDPK2* was helpful for the expression of genes related to toluene metabolism, thus improving the toluene absorption capacity of the plant.

## Figures and Tables

**Table 1:** Summary of the *A. pusilla* leaf transcriptome.

Samples	1-1	1-2	2-1	2-2
Total raw reads	137,518,750	165,188,286	144,957,778	138,077,056
Total trimmed reads	135,591,762	162,623,598	142,958,464	136,257,376
Q20 (%)	98.90	98.78	98.82	98.86
Q30 (%)	96.03	95.69	95.78	95.87
GC (%)	50.30	49.99	50.52	49.37
Mapped reads	100,780,248 (74.33%)	117,220,734 (72.08%)	110,070,246 (76.99%)	97,991,310 (71.92%)
Unmapped reads	34,811,514 (25.67%)	45,402,864 (27.92%)	32,888,218 (23.01%)	38,266,066 (28.08%)

1-1 and 1-2: control samples (two independent biological replicates). 2-1 and 2-2: toluene treated sample (two independent biological replicates).

**Table 2:** The number of DEGs in toluene treated and untreated *A. pusilla* plants.

DEGs Set	Total DEGs	KEGG DEGs	GO DEGs			
			Total	Biological Processes	Cellular Components	Molecular Functions
All DEGs	4101	3101	2854	2393	2525	2547
Up regulation	2100 (51.2%)	1610 (51.9%)	1456 (51.0%)	1234 (51.6%)	1262 (50.0%)	1315 (51.6%)
Down regulation	2001 (48.8%)	1491 (48.1%)	1398 (49.0%)	1159 (48.4%)	1263 (50.0%)	1232 (48.4%)

## Data Availability

The complete RNA-seq data were submitted to GEO. The accession number is GSE172268 (https://www.ncbi.nlm.nih.gov/geo/query/acc.cgi?acc=GSE172268).

## Supplementary Materials

The following are available online at https://www.mdpi.com/article/10.3390/plants10051011/s1, Figure S1: GO functional analysis of DEGs identified in toluene treated and untreated *A. pusilla*, Figure S2: KEGG analysis DEGs in ribosome after toluene treatment in *A. pusilla*, Figure S3: KEGG analysis DEGs in endocytosis after toluene treatment in *A. pusilla*, Table S1: Primer sequences for metabolic pathways and reference genes used in this study, Table S2: Assembly statistics and statistics of clustered contigs (unigene) in *A. pusilla* leaf transcriptome, Table S3 (Additional file 1): GO functional classification analysis on each type of unigenes, Table S4 (Additional file 1): *A. pusilla* DEGs matched with Arabidopsis gene sequence.

## References

[1] Flowers L., Boyes W., Foster S., Gelhaus M., Hogan K., Marcus A., McClure P., Osier M., Ronney A. (2005). Toxicological Review of Toluene (CAS No. 108–88-3).

[2] Muccee F., Ejaz S., Riaz N. (2019). Toluene degradation via a unique metabolic route in indigenous bacterial species. Arch. Microbiol..

[3] Becker J.M., Neal M.W. (1990). Drinking Water Criteria Document for Toluene.

[4] De Bortoli M., Knöppel H., Pecchio E., Peil A., Rogora L., Schauenburg H., Schlitt H., Vissers H. (1986). Concentrations of selected organic pollutants in indoor and outdoor air in northern Italy. Environ. Int..

[5] Faradisha J., Tualeka A.R., Widajati N. (2019). Analysis of Correlation between Toluene Exposure and Health Risk Characterization on Printing Worker of Plastic Bags Industry. Indian J. Public Health Res. Dev..

[6] Shih H.-T., Yu C.-L., Wu M.-T., Liu C.-S., Tsai C.-H., Hung D.-Z., Wu C.-S., Kuo H.-W. (2011). Subclinical abnormalities in workers with continuous low-level toluene exposure. Toxicol. Ind. Health.

[7] Singhai A. (2013). Toluene-induced acute lung injury. Med. J. DY Patil Univ..

[8] Irga P.J., Pettit T., Irga R.F., Paull N.J., Douglas A.N., Torpy F.R. (2019). Does plant species selection in functional active green walls influence VOC phytoremediation efficiency?. Environ. Sci. Pollut. Res..

[9] Kim K.J., Khalekuzzaman M., Suh J.N., Kim H.J., Shagol C., Kim H.-H., Kim H.J. (2018). Phytoremediation of volatile organic compounds by indoor plants: A review. Hortic. Environ. Biotechnol..

[10] Hörmann V., Brenske K.-R., Ulrichs C. (2018). Assessment of filtration efficiency and physiological responses of selected plant species to indoor air pollutants (toluene and 2-ethylhexanol) under chamber conditions. Environ. Sci. Pollut. Res..

[11] Kim K.J., Yoo E.H., Jeong M.I., Song J.S., Lee S.Y., Kays S.J. (2011). Changes in the phytoremediation potential of indoor plants with exposure to toluene. HortScience.

[12] Sriprapat W., Suksabye P., Areephak S., Klantup P., Waraha A., Sawattan A., Thiravetyan P. (2014). Uptake of toluene and ethylbenzene by plants: Removal of volatile indoor air contaminants. Ecotoxicol. Environ. Saf..

[13] Group A.P. (2009). An update of the Angiosperm Phylogeny Group classification for the orders and families of flowering plants: APG III. Bot. J. Linn. Soc..

[14] Ahn C.H., Kim N.-S., Shin J.Y., Lee Y.A., Kim K.J., Kim J.H., Park P.M., An H.R., Kim Y.-J., Kim W.H. (2020). Enhanced detoxification of exogenous toluene gas in transgenic Ardisia pusilla expressing AtNDPK2 gene. Hortic. Environ. Biotechnol..

[15] Song J.E. (2012). A study on the reduction of volatile organic compounds by Fatsia japonica and Ardisia pusilla. J. Korea Inst. Ecol. Archit. Environ..

[16] Kim Y.-H., Lim S., Yang K.-S., Kim C.Y., Kwon S.-Y., Lee H.-S., Wang X., Zhou Z., Ma D., Yun D.-J. (2009). Expression of Arabidopsis NDPK2 increases antioxidant enzyme activities and enhances tolerance to multiple environmental stresses in transgenic sweetpotato plants. Mol. Breed..

[17] Wang Z., Li H., Ke Q., Jeong J.C., Lee H.-S., Xu B., Deng X.-P., Lim Y.P., Kwak S.-S. (2014). Transgenic alfalfa plants expressing AtNDPK2 exhibit increased growth and tolerance to abiotic stresses. Plant Physiol. Biochem..

[18] Zhang J., Movahedi A., Sang M., Wei Z., Xu J., Wang X., Wu X., Wang M., Yin T., Zhuge Q. (2017). Functional analyses of NDPK2 in Populus trichocarpa and overexpression of PtNDPK2 enhances growth and tolerance to abiotic stresses in transgenic poplar. Plant Physiol. Biochem..

[19] Ramya M., Park P.H., Chuang Y.-C., Kwon O.K., An H.R., Park P.M., Baek Y.S., Kang B.-C., Tsai W.-C., Chen H.-H. (2019). RNA sequencing analysis of Cymbidium goeringii identifies floral scent biosynthesis related genes. BMC Plant Biol..

[20] Chen J., Le X.C., Zhu L. (2019). Metabolomics and transcriptomics reveal defense mechanism of rice (Oryza sativa) grains under stress of 2, 2’, 4, 4’-tetrabromodiphenyl ether. Environ. Int..

[21] Langmead B., Trapnell C., Pop M., Salzberg S.L. (2009). Ultrafast and memory-efficient alignment of short DNA sequences to the human genome. Genome Biol..

[22] Li B., Dewey C.N. (2011). RSEM: Accurate transcript quantification from RNA-Seq data with or without a reference genome. BMC Bioinform..

[23] Ding Y., Xue X., Liu Z., Ye Y., Xiao P., Pu Y., Guan W., Mwacharo J.M., Ma Y., Zhao Q. (2020). Expression Profiling and Functional Characterization of miR-26a and miR-130a in Regulating Zhongwei Goat Hair Development via the TGF-β/SMAD Pathway. Int. J. Mol. Sci..

[24] Xia Z., Xu H., Zhai J., Li D., Luo H., He C., Huang X. (2011). RNA-Seq analysis and de novo transcriptome assembly of Hevea brasiliensis. Plant Mol. Biol..

[25] Altermann E., Klaenhammer T.R. (2005). PathwayVoyager: Pathway mapping using the Kyoto Encyclopedia of Genes and Genomes (KEGG) database. BMC Genom..

[26] Durmishidze S. (1975). Cleavage of the Aromatic Ring of Some Exogenous Compounds in Plants.

[27] Ugrekhelidze D.S. (1976). Metabolizm Ekzogennykh Alkanov i Aromaticheskikh Uglevodorodov v Rasteniyakh (Metabolism of Exogenous Alkanes and Aromatic Hydrocarbons in Plants).

[28] Ugrekhelidze D., Korte F., Kvesitadze G. (1997). Uptake and transformation of benzene and toluene by plant leaves. Ecotoxicol. Environ. Saf..

[29] Singh K.B., Foley R.C., Oñate-Sánchez L. (2002). Transcription factors in plant defense and stress responses. Curr. Opin. Plant Biol..

[30] Zhang J.Z. (2003). Overexpression analysis of plant transcription factors. Curr. Opin. Plant Biol..

[31] Ma D., Constabel C.P. (2019). MYB repressors as regulators of phenylpropanoid metabolism in plants. Trends Plant Sci..

[32] Ampomah-Dwamena C., Thrimawithana A.H., Dejnoprat S., Lewis D., Espley R.V., Allan A.C. (2019). A kiwifruit (Actinidia deliciosa) R2R3-MYB transcription factor modulates chlorophyll and carotenoid accumulation. New Phytol..

[33] Meng Y., Wang Z., Wang Y., Wang C., Zhu B., Liu H., Ji W., Wen J., Chu C., Tadege M. (2019). The MYB activator WHITE PETAL1 associates with MtTT8 and MtWD40-1 to regulate carotenoid-derived flower pigmentation in Medicago truncatula. Plant Cell.

[34] Li B., Fan R., Guo S., Wang P., Zhu X., Fan Y., Chen Y., He K., Kumar A., Shi J. (2019). The Arabidopsis MYB transcription factor, MYB111 modulates salt responses by regulating flavonoid biosynthesis. Environ. Exp. Bot..

[35] Wang X.C., Wu J., Guan M.L., Zhao C.H., Geng P., Zhao Q. (2020). Arabidopsis MYB4 plays dual roles in flavonoid biosynthesis. Plant J..

[36] Geng P., Zhang S., Liu J., Zhao C., Wu J., Cao Y., Fu C., Han X., He H., Zhao Q. (2020). MYB20, MYB42, MYB43, and MYB85 regulate phenylalanine and lignin biosynthesis during secondary cell wall formation. Plant Physiol..

[37] Lee S.B., Kim H.U., Suh M.C. (2016). MYB94 and MYB96 additively activate cuticular wax biosynthesis in Arabidopsis. Plant Cell Physiol..

[38] Wang S.-S., Song Z.-B., Sun Z., Zhang J., Mei Y., Nian H.-J., Li K.-Z., Chen L.-M. (2012). Physiological and transcriptional analysis of the effects of formaldehyde exposure on Arabidopsis thaliana. Acta Physiol. Plant..

[39] Caarls L., Van der Does D., Hickman R., Jansen W., Verk M.C.V., Proietti S., Lorenzo O., Solano R., Pieterse C.M., Van Wees S. (2017). Assessing the role of ETHYLENE RESPONSE FACTOR transcriptional repressors in salicylic acid-mediated suppression of jasmonic acid-responsive genes. Plant Cell Physiol..

[40] Fujimoto S.Y., Ohta M., Usui A., Shinshi H., Ohme-Takagi M. (2000). Arabidopsis ethylene-responsive element binding factors act as transcriptional activators or repressors of GCC box–mediated gene expression. Plant Cell.

[41] Son G.H., Wan J., Kim H.J., Nguyen X.C., Chung W.S., Hong J.C., Stacey G. (2012). Ethylene-responsive element-binding factor 5, ERF5, is involved in chitin-induced innate immunity response. Mol. Plant Microbe Interact..

[42] Wehner N., Hartmann L., Ehlert A., Böttner S., Oñate-Sánchez L., Dröge-Laser W. (2011). High-throughput protoplast transactivation (PTA) system for the analysis of Arabidopsis transcription factor function. Plant J..

[43] Yang Z., Tian L., Latoszek-Green M., Brown D., Wu K. (2005). Arabidopsis ERF4 is a transcriptional repressor capable of modulating ethylene and abscisic acid responses. Plant Mol. Biol..

[44] Chen W.-H., Li P.-F., Chen M.-K., Lee Y.-I., Yang C.-H. (2015). FOREVER YOUNG FLOWER negatively regulates ethylene response DNA-binding factors by activating an ethylene-responsive factor to control Arabidopsis floral organ senescence and abscission. Plant Physiol..

[45] Osnato M., Cereijo U., Sala J., Matías-Hernández L., Aguilar-Jaramillo A.E., Rodríguez-Goberna M.R., Riechmann J.L., Rodríguez-Concepción M., Pelaz S. (2021). The floral repressors TEMPRANILLO1 and 2 modulate salt tolerance by regulating hormonal components and photo-protection in Arabidopsis. Plant J..

[46] Fu M., Kang H.K., Son S.-H., Kim S.-K., Nam K.H. (2014). A subset of Arabidopsis RAV transcription factors modulates drought and salt stress responses independent of ABA. Plant Cell Physiol..

[47] Hsu F.-C., Chou M.-Y., Chou S.-J., Li Y.-R., Peng H.-P., Shih M.-C. (2013). Submergence confers immunity mediated by the WRKY22 transcription factor in Arabidopsis. Plant Cell.

[48] Bao W., Wang X., Chen M., Chai T., Wang H. (2018). A WRKY transcription factor, PcWRKY33, from Polygonum cuspidatum reduces salt tolerance in transgenic Arabidopsis thaliana. Plant Cell Rep..

[49] Snider N.T., Altshuler P.J., Omary M.B. (2015). Modulation of cytoskeletal dynamics by mammalian nucleoside diphosphate kinase (NDPK) proteins. Naunyn-Schmiedeberg’s Arch. Pharmacol..

[50] Liu H., Weisman D., Tang L., Tan L., Zhang W.-K., Wang Z.-H., Huang Y.-H., Lin W.-X., Liu X.-M., Colón-Carmona A. (2015). Stress signaling in response to polycyclic aromatic hydrocarbon exposure in Arabidopsis thaliana involves a nucleoside diphosphate kinase, NDPK-3. Planta.

[51] Luzarowski M., Kosmacz M., Sokolowska E., Jasińska W., Willmitzer L., Veyel D., Skirycz A. (2017). Affinity purification with metabolomic and proteomic analysis unravels diverse roles of nucleoside diphosphate kinases. J. Exp. Bot..

[52] Bolger A.M., Lohse M., Usadel B. (2014). Trimmomatic: A flexible trimmer for Illumina sequence data. Bioinformatics.

[53] Grabherr M.G., Haas B.J., Yassour M., Levin J.Z., Thompson D.A., Amit I., Adiconis X., Fan L., Raychowdhury R., Zeng Q. (2011). Trinity: Reconstructing a full-length transcriptome without a genome from RNA-Seq data. Nat. Biotechnol..

[54] Li W., Godzik A. (2006). Cd-hit: A fast program for clustering and comparing large sets of protein or nucleotide sequences. Bioinformatics.

[55] Fu L., Niu B., Zhu Z., Wu S., Li W. (2012). CD-HIT: Accelerated for clustering the next-generation sequencing data. Bioinformatics.

[56] Buchfink B., Xie C., Huson D.H. (2015). Fast and sensitive protein alignment using DIAMOND. Nat. Methods.

[57] Li B., Ruotti V., Stewart R.M., Thomson J.A., Dewey C.N. (2010). RNA-Seq gene expression estimation with read mapping uncertainty. Bioinformatics.

[58] Haas B.J., Papanicolaou A., Yassour M., Grabherr M., Blood P.D., Bowden J., Couger M.B., Eccles D., Li B., Lieber M. (2013). De novo transcript sequence reconstruction from RNA-seq using the Trinity platform for reference generation and analysis. Nat. Protoc..

[59] Consortium G.O. (2008). The gene ontology project in 2008. Nucleic Acids Res..

[60] Kanehisa M., Araki M., Goto S., Hattori M., Hirakawa M., Itoh M., Katayama T., Kawashima S., Okuda S., Tokimatsu T. (2007). KEGG for linking genomes to life and the environment. Nucleic Acids Res..

