# Supplementary material for: Transcriptomic Analysis for the Identification of Metabolic Pathway Genes Related to Toluene Response in Ardisia pusilla
Source: Plants (Basel). 2021 May 19;10(5):1011. doi: 10.3390/plants10051011 (PMC8159097; doi:10.3390/plants10051011)
Supplement: Supplementary file 1 [file plants-10-01011-s001.zip › Supplementary tables and figures-Junping Xu.pdf]

Table S1. Primer sequences for metabolic pathways and reference genes used in this study.

Gene name	GenBank ID		Primer Sequence(5' to 3')
OMT1	NM_124796.4	Forward	CCCTCTTCGCCATGCAACTA
		Reverse	AGCGATCTCGGTAGGAGACA
APG1	NM_116206.3	Forward	ATGCAAGATCGTCGAGGGAG
		Reverse	AGAAGGGGTTGTTGACAGGC
HAI	NM_127453.4	Forward	GGTCTTAGGTCGTTGGCAGT
		Reverse	CTGTCGTGTCTTGGAGGGTC
GAPCP1	NM_106601.4	Forward	ACGGCTACAGAAGTGCCATC
		Reverse	ACCAACCTTCCAATCCGACC
YUC4	NM_180477.2	Forward	GCGAGCTTCCTCTTATGCCT
		Reverse	ACCGTCTTGCGTCTTCACAT
MDH	NM_114620.3	Forward	GTGCTGCTGGTGGTATAGGT
		Reverse	GTTAGCCCTCACAACATCCA
EFE	NM_100380.4	Forward	GCATCATCCTCCTCTTCCA
		Reverse	GGAGGAACATCGACCCACTC
TIM	NM_127687.4	Forward	ACCGCGTCAATTCCTCTACC
		Reverse	GCATCAGCAAACGCCTTCAG
NIR	NM_127123.3	Forward	ATTGGCAGATCCGTGGTGTT
		Reverse	CCCAAGCATCAAGAGGAATCG
At4G17260	NM_117832.4	Forward	AACTCTCTGGCTTTCCGGTG
		Reverse	AATGCCACTGAGCTGTCTCC
Actin	XM_009601581.3	Forward	TGCTGATCGTATGAGCAAGGAA
		Reverse	GGTGGAGCAACAACCTTAATCTTC

OMT1: *O-methyltransferase 1*; *APG1*: *S-adenosyl-l-methylionine-dependent methyltransferases superfamily protein*; *HAI*: *H [+] - ATPase 1*; *GAPCP1*: *glyceraldehyde-3-phosphate dehydrogenation of plastid 1*; *YUC4*: *flavin binding monooxygenase family protein*; *MDH*: *malate dehydrogenase*; *EFE*: *ethylene forming enzyme*; *TIM*: *triosephosphate isomerase*; *NIR*: *nitrite reductase 1*; AT4G17260: *lactate/malate dehydrogenase family protein*.

Table S2. Assembly statistics and statistics of clustered contigs (unigene) in *A. pusilla* leaf transcriptome.

Assembly	Merge		
	All transcript contigs	Only longest isoform per 'gene'	Clustered Contig 'Unigene'
Total trinity 'genes'	99,322	99,322	88,444
Total trinity transcripts	135,095	99,322	88,444
Percent GC (%)	42.85	42.92	42.71
N50	1,137	955	1,049
Maximum contig length (bp)	17,716	17,716	17,716
Minimum contig length (bp)	201	201	201
Median contig length (bp)	397	340	350
Average contig length (bp)	694.06	609.44	637
Total assembled bases(bp)	93,764,604	60,531,284	56,354,554

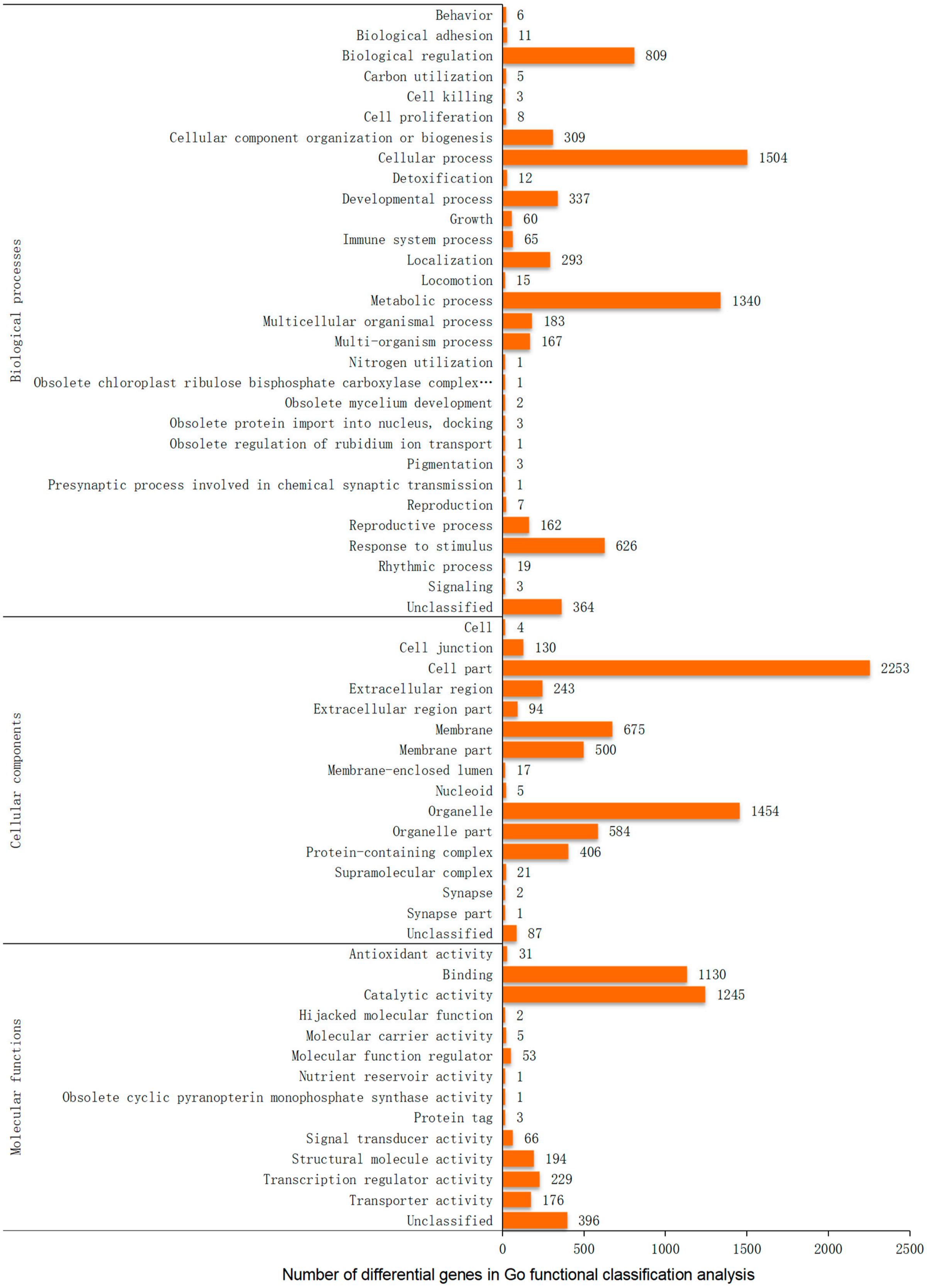

Figure S1. GO functional analysis of DEGs identified in toluene treated and untreated *A. pusilla*.

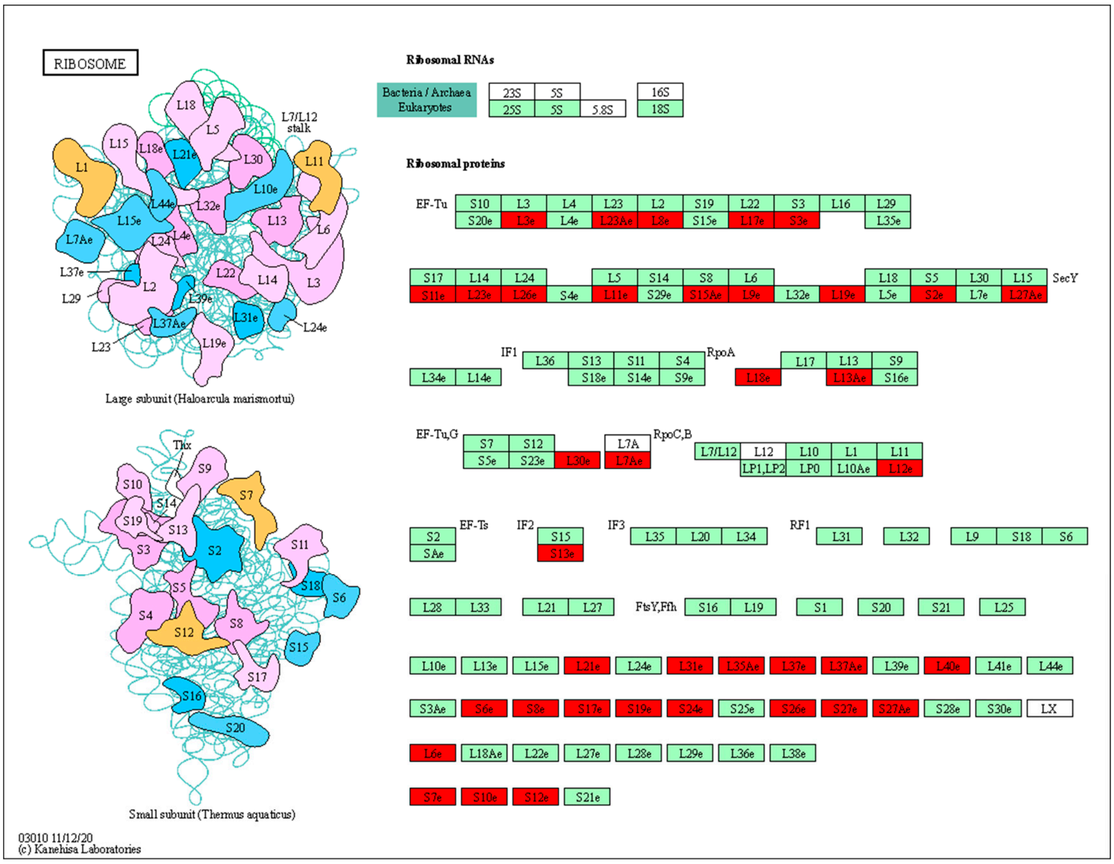

Figure S2. KEGG analysis DEGs in ribosome after toluene treatment in *A. pusilla.* Red bars: up-regulation; blue bars: down-regulation; green bars: represent organism-specific pathways, and indicate that map objects exist and are linked to corresponding entries.

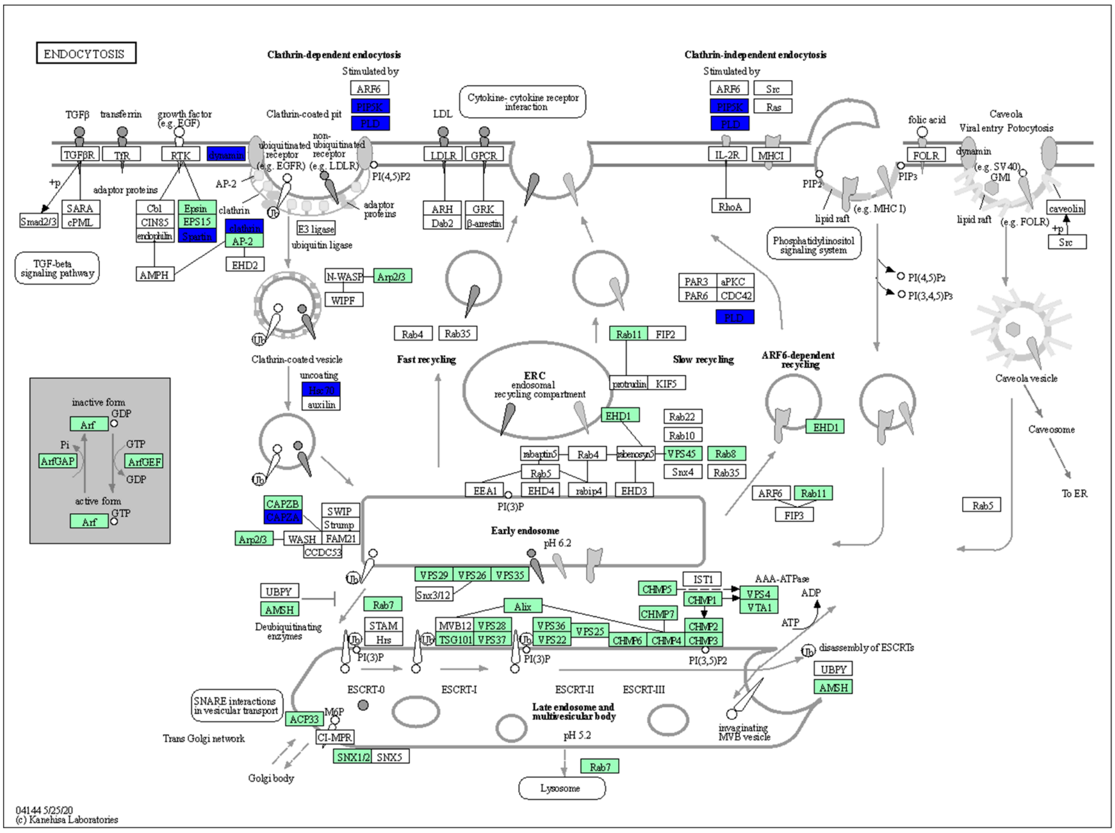

Figure S3. KEGG analysis DEGs in endocytosis after toluene treatment in *A. pusilla.* Red bars: up-regulation; blue bars: down-regulation; green bars: represent organism-specific pathways, and indicate that map objects exist and are linked to corresponding entries.
